# Supplementary material for: COMBSecretomics: A pragmatic methodological framework for higher-order drug combination analysis using secretomics
Source: PLoS One. 2020 May 14;15(5):e0232989. doi: 10.1371/journal.pone.0232989 (PMC7224510; doi:10.1371/journal.pone.0232989)
Supplement: S1 File — (PDF) [file pone.0232989.s001.pdf]

Supporting File 1

COMBSecretomics: a pragmatic methodological framework for higher-order drug combination analysis using secretomics

Efthymia Chantzi *[1], Michael Neidlin[2], George A. Macheras[3], Leonidas G. Alexopoulos[2], and Mats G. Gustafsson*[1]

[1]Department of Medical Sciences, Cancer Pharmacology and Computational Medicine, Uppsala University, Uppsala, Sweden
[2]Department of Mechanical Engineering, National Technical University of Athens, Athens, Greece
[3]4th Orthopedic Department, General Hospital KAT, Athens, Greece

Contents

1 Quality control explained **1**
1.1 Blank filtering . . . 1
1.1.1 Imputation . . . 2
1.1.2 Coefficient of variation . . . 2

2 Normalization of protein release differences **4**

3 Example raw data file **15**

4 User-defined inputs **16**

List of Figures

1 Blank filtering . . . 1
2 Pre-imputation QC . . . 2
3 Intra-plate coefficient of variation . . . 3
4 Median of intra-plate replicate measurements . . . 3
5 Therapeutic need for unstimulated cells (Q1) . . . 4
6 Modulation capacity for unstimulated cells (Q2) . . . 4
7 Restoration capacity for unstimulated cells (Q3) . . . 5
8 Therapeutic need for stimulated cells (Q1) . . . 5
9 Modulation capacity for $S1$-stimulated cells (Q2) . . . 6
10 Modulation capacity for $S2$-stimulated cells (Q2) . . . 6
11 Modulation capacity for $S3$-stimulated cells (Q2) . . . 7
12 Restoration capacity for $S1$-stimulated cells (Q3) . . . 7
13 Restoration capacity for $S2$-stimulated cells (Q3) . . . 8
14 Restoration capacity for $S3$-stimulated cells (Q3) . . . 8
15 Top-down hierarchical clustering for $S2$-stimulated cells . . . 9
16 Top-down hierarchical clustering for $S3$-stimulated cells . . . 10
17 Top-down hierarchical clustering for unstimulated cells . . . 11
18 Top-down hierarchical clustering for $S1$-stimulated cells and exhaustive subset search . . . 12

*correspondence: efthymia.chantzi@medsci.uu.se, mats.gustafsson@medsci.uu.se

19 GHSA for $S2$-stimulated cells 13
20 GHSA for $S3$-stimulated cells 14
21 GHSA for unstimulated cells 15

1 Quality control explained

Quality control (QC) procedures include pre-processing of the collected raw measurement values per experimental plate/batch, in order to eliminate noise and exclude outliers that may trigger misinterpretations. The sequential QC steps employed currently by COMBSecretomics are described below in different subsections.

1.1 Blank filtering

Experimental wells where the raw measurement values are smaller than the 95th percentile of the corresponding blank wells are set to missing values. This is in order to be able to perform imputation, as explained in the next step, instead of directly throwing out data. However, imputation may also lead to biased results, especially if too many values have to be imputed (Fig. S1). For this reason, COMBSecretomics allows a limited number of imputations (Fig. S2). Specifically, a particular protein k (i.e., a column in the $N \times d$ data matrix) is kept only if less than a user-defined percentage of all measurements correspond to missing values. Subsequently, a particular cell state (i.e., a row in the $N \times d$ data matrix) is kept only if less than the aforementioned user-defined percentage of all measurements correspond to missing values. The higher this threshold, the more imputations are going to be performed. For our case study, this threshold was set to 15%.

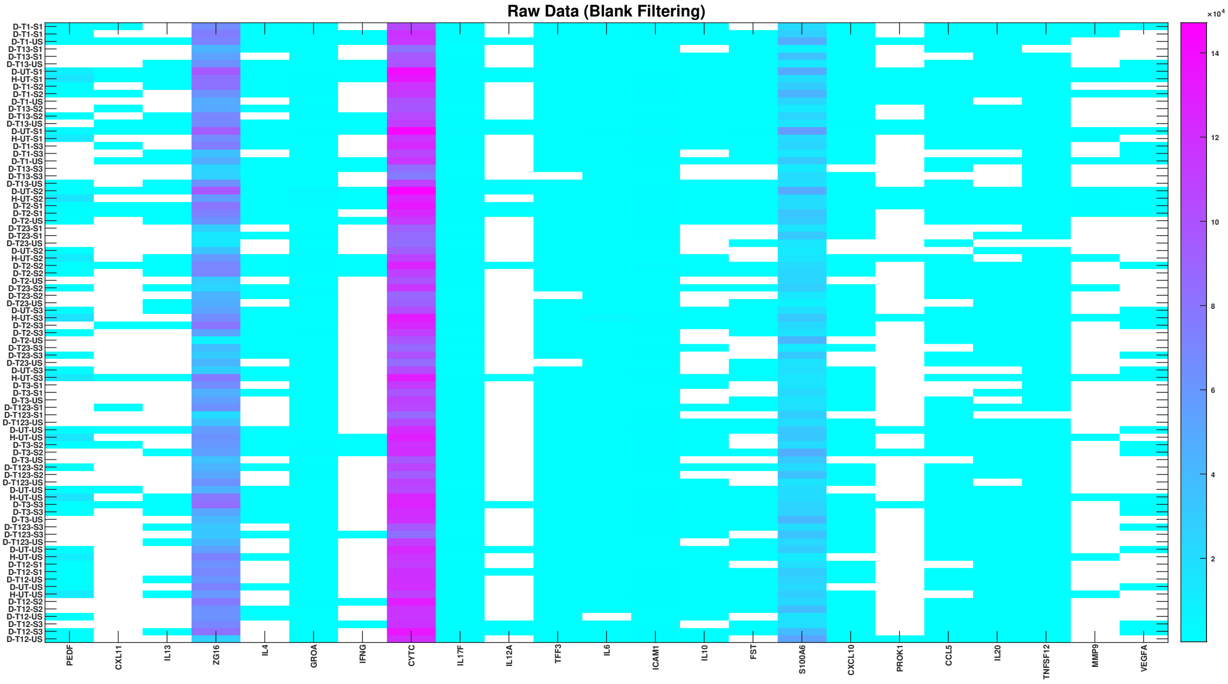

Figure S1: Blank filtering. Raw data values after blank filtering. All measurements that have been excluded are set to missing values and displayed in white. The measured protein panel is shown in the horizontal axis, while all different experimental wells/cell states are shown in the vertical axis.

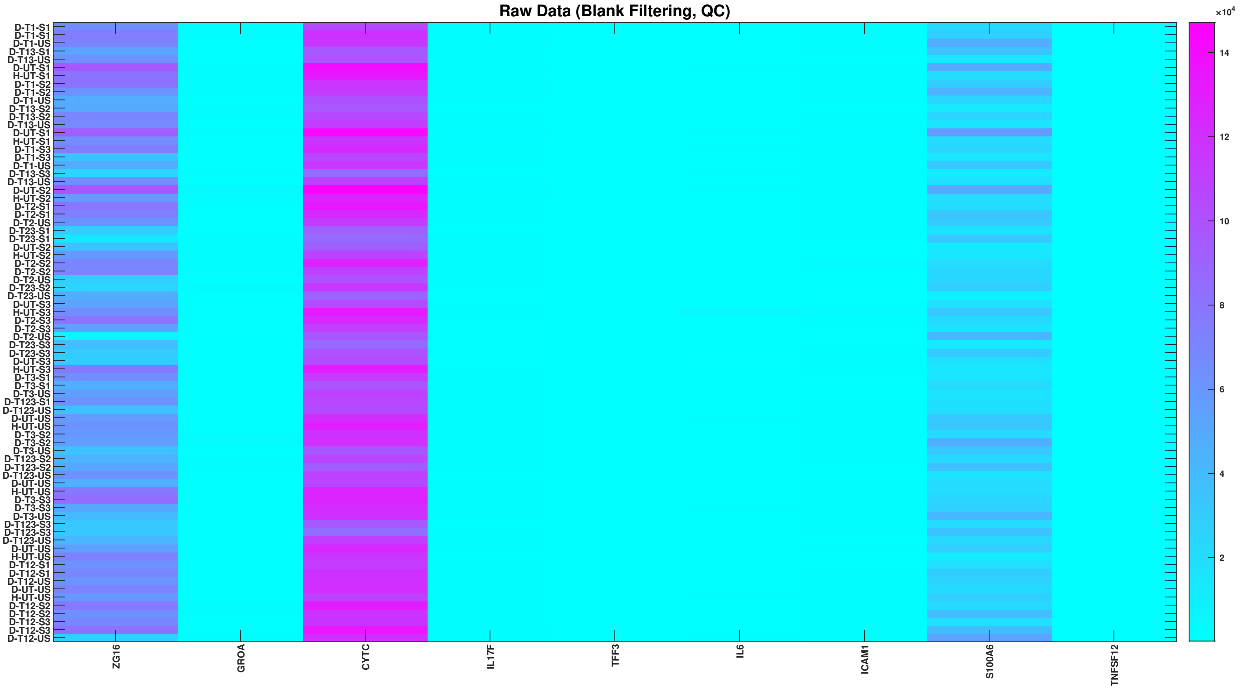

Figure S2: Pre-imputation QC. Raw data values after excluding proteins and experimental wells with a high number of missing values due to blank filtering. This step aims at eliminating biased results emerging from imputing too many missing values. As shown, after this quality control step, 9 out of 23 proteins are kept.

1.1.1 Imputation

Missing data (as obtained from the previous step) are imputed in the horizontal direction (i.e., the closest row in the raw $N \times d$ data matrix) using the euclidean distance as metric. For this task, the function *knnimpute* is used, as implemented in MATLAB R2019b, by employing default settings. In terms of our case study, no imputations were needed as shown in Fig. S2.

1.1.2 Coefficient of variation

A last QC step is employed in order to ensure that there is limited technical variability between intra-plate replicate measurement values for the protein releases (i.e., columns in the $N \times d$ data matrix). More specifically, for a particular protein k, the coefficient of variation for all intra-plate replicate wells is calculated. Since there are up to 6 different cell states, each protein k has up to 6 different coefficients of variation, one for each state. The protein d_i is kept for further analysis only if the median across all these coefficients of variation is below a user-defined threshold. For this particular case study, this threshold was set to 25% (Fig. S3).

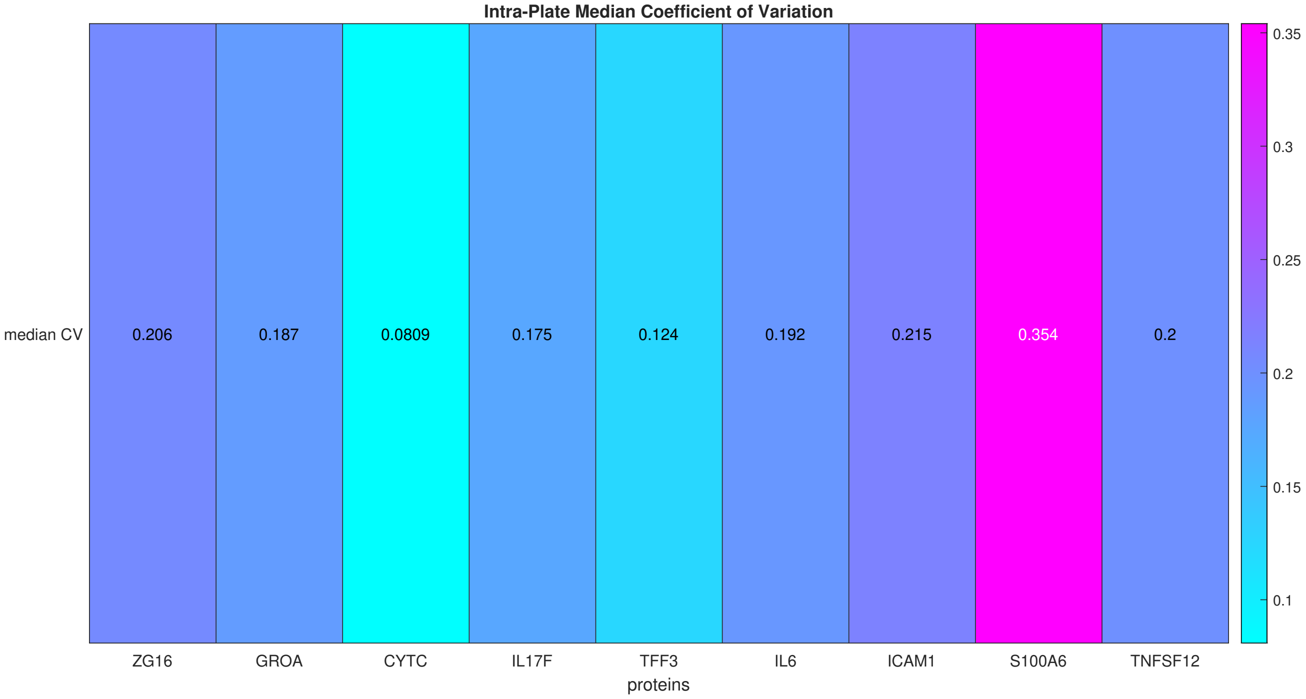

Figure S3: Intra-plate coefficient of variation. Median coefficient of variation for the 9 proteins remained after all previous quality control steps. Given the cut-off threshold of 25%, S100A6 was also excluded from further analyses.

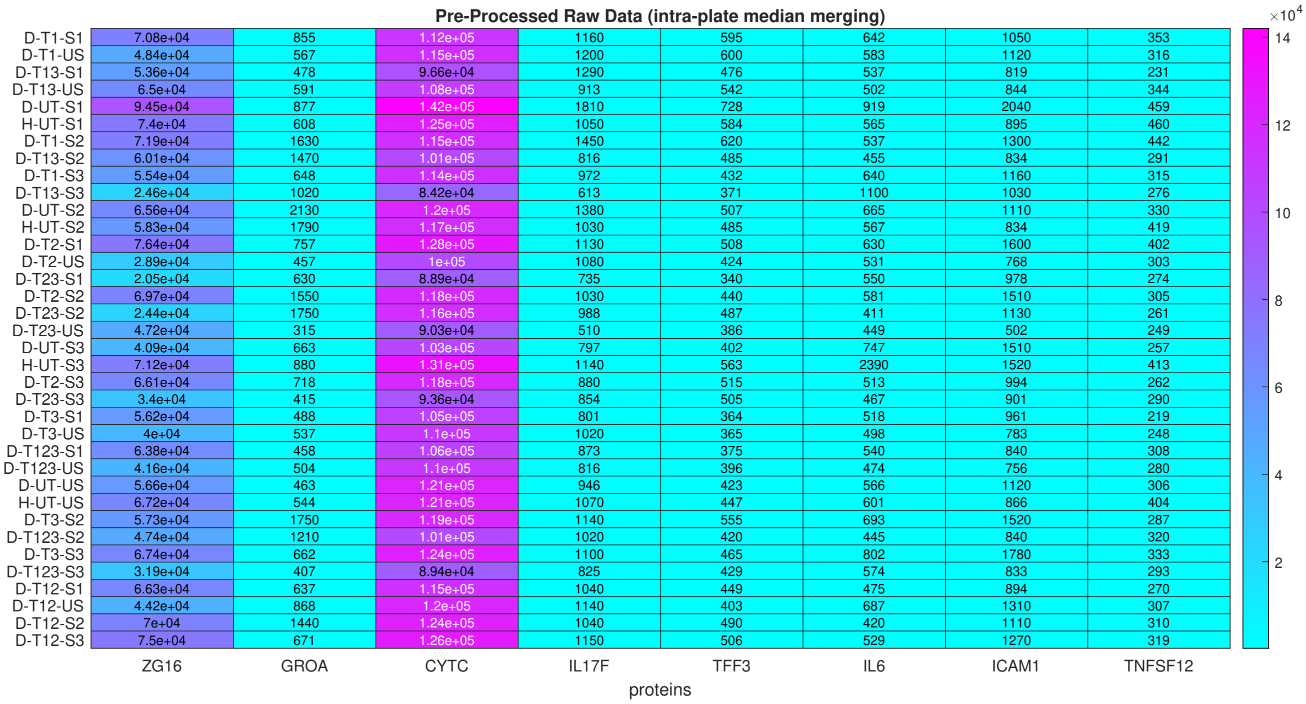

	ZG16	GROA	CYTC	IL17F	TFF3	IL6	ICAM1	TNFSF12
D-T1-S1	7.08e+04	855	1.12e+05	1160	595	642	1050	353
D-T1-US	4.84e+04	567	1.15e+05	1200	600	583	1120	316
D-T13-S1	5.36e+04	478	9.66e+04	1290	476	537	819	231
D-T13-US	6.5e+04	591	1.08e+05	913	542	502	844	344
D-UT-S1	9.45e+04	877	1.42e+05	1810	728	919	2040	459
H-UT-S1	7.4e+04	608	1.25e+05	1050	584	565	895	460
D-T1-S2	7.19e+04	1630	1.15e+05	1450	620	537	1300	442
D-T13-S2	6.01e+04	1470	1.01e+05	816	485	455	834	291
D-T1-S3	5.54e+04	648	1.14e+05	972	432	640	1160	315
D-T13-S3	2.46e+04	1020	8.42e+04	613	371	1100	1030	276
D-UT-S2	6.56e+04	2130	1.2e+05	1380	507	665	1110	330
H-UT-S2	5.83e+04	1790	1.17e+05	1030	485	567	834	419
D-T2-S1	7.64e+04	757	1.28e+05	1130	508	630	1600	402
D-T2-US	2.89e+04	457	1e+05	1080	424	531	768	303
D-T23-S1	2.05e+04	630	8.89e+04	735	340	550	978	274
D-T2-S2	6.97e+04	1550	1.18e+05	1030	440	581	1510	305
D-T23-S2	2.44e+04	1750	1.16e+05	988	487	411	1130	261
D-T23-US	4.72e+04	315	9.03e+04	510	386	449	502	249
D-UT-S3	4.09e+04	663	1.03e+05	797	402	747	1510	257
H-UT-S3	7.12e+04	880	1.31e+05	1140	563	2390	1520	413
D-T2-S3	6.61e+04	718	1.18e+05	880	515	513	994	262
D-T23-S3	3.4e+04	415	9.36e+04	854	505	467	901	290
D-T3-S1	5.62e+04	488	1.05e+05	801	364	518	961	219
D-T3-US	4e+04	537	1.1e+05	1020	365	498	783	248
D-T123-S1	6.38e+04	458	1.06e+05	873	375	540	840	308
D-T123-US	4.16e+04	504	1.1e+05	816	396	474	756	280
D-UT-US	5.66e+04	463	1.21e+05	946	423	566	1120	306
H-UT-US	6.72e+04	544	1.21e+05	1070	447	601	866	404
D-T3-S2	5.73e+04	1750	1.19e+05	1140	555	693	1520	287
D-T123-S2	4.74e+04	1210	1.01e+05	1020	420	445	840	320
D-T3-S3	6.74e+04	662	1.24e+05	1100	465	802	1780	333
D-T123-S3	3.19e+04	407	8.94e+04	825	429	574	833	293
D-T12-S1	6.63e+04	637	1.15e+05	1040	449	475	894	270
D-T12-US	4.42e+04	868	1.2e+05	1140	403	687	1310	307
D-T12-S2	7e+04	1440	1.24e+05	1040	490	420	1110	310
D-T12-S3	7.5e+04	671	1.26e+05	1150	506	529	1270	319

Figure S4: Median of intra-plate replicate measurements. The measured proteins are shown in the x-axis, while the different cell states are illustrated in the y-axis. UT and US stand for untreated and unstimulated cells respectively. 8 out of 23 proteins were used for further analysis (ZG16, GROA, CYTC, IL17F, TFF3, IL6, ICAM1, S100A6, TNFSF12).

2 Normalization of protein release differences

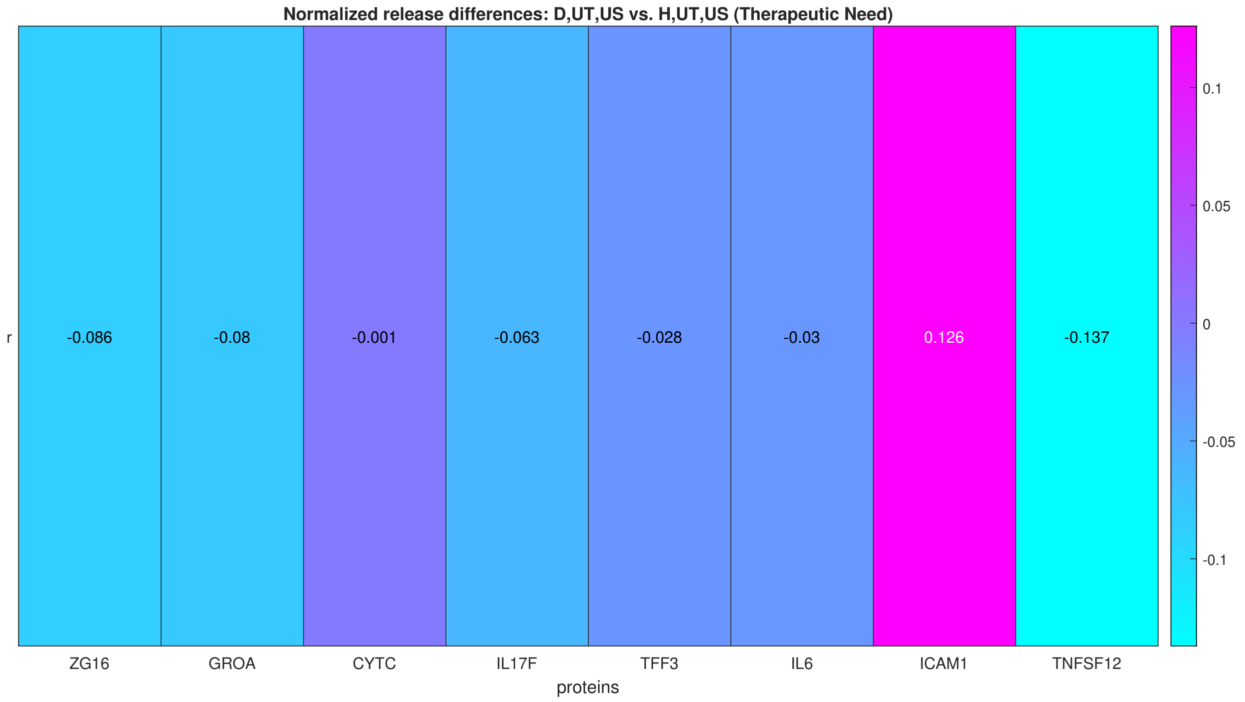

Figure S5: Normalized release differences between D,untreated (UT),unstimulated (US) and H,untreated (UT),unstimulated cells (US). Small differences in protein releases are observed for all apart from two proteins. In particular, the release levels of ICAM1 and TNFSF12 seem to increase ≈ 13% and drop ≈ 14% respectively in D compared to H cells.

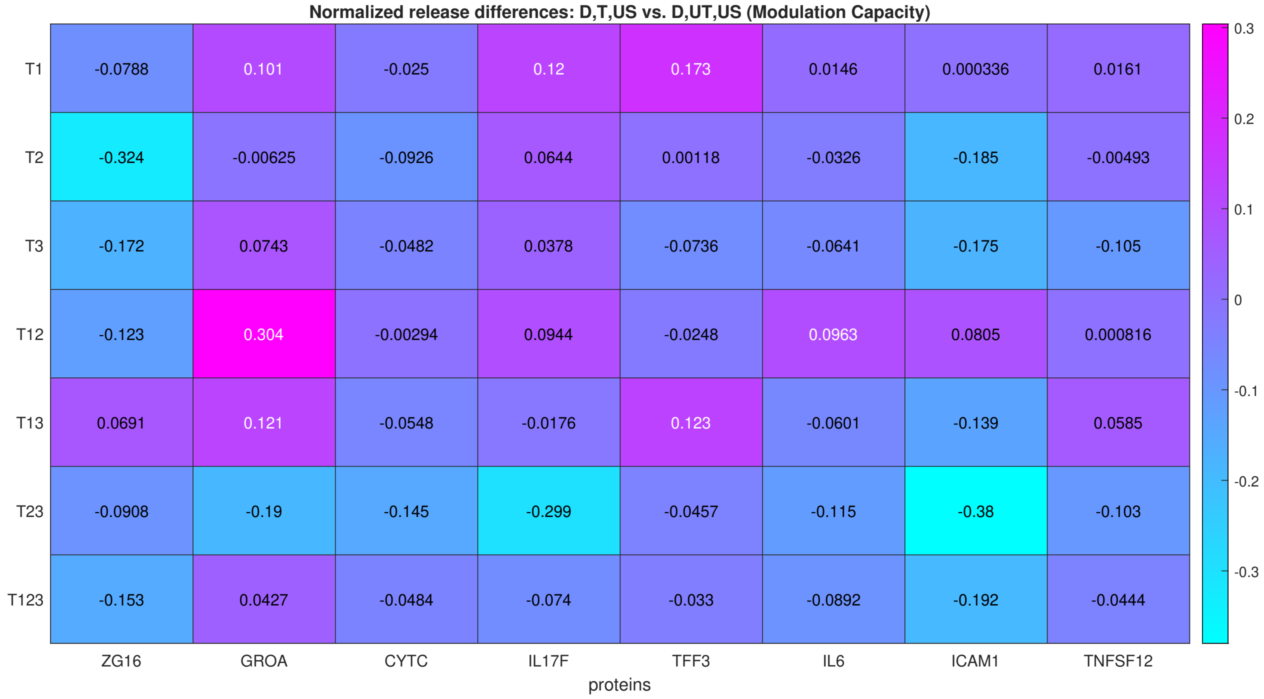

Figure S6: Normalized release differences between D,treated (T),unstimulated (US) and D,untreated (UT),unstimulated cells (US). A particular row shows how much a particular treatment T_x has affected the protein releases of D cells. For example, T_{12} seems to have resulted in ≈ 12% less release of ZG16 and ≈ 30% more release of GROA, while the remaining 6 proteins seems to be relatively unaffected.

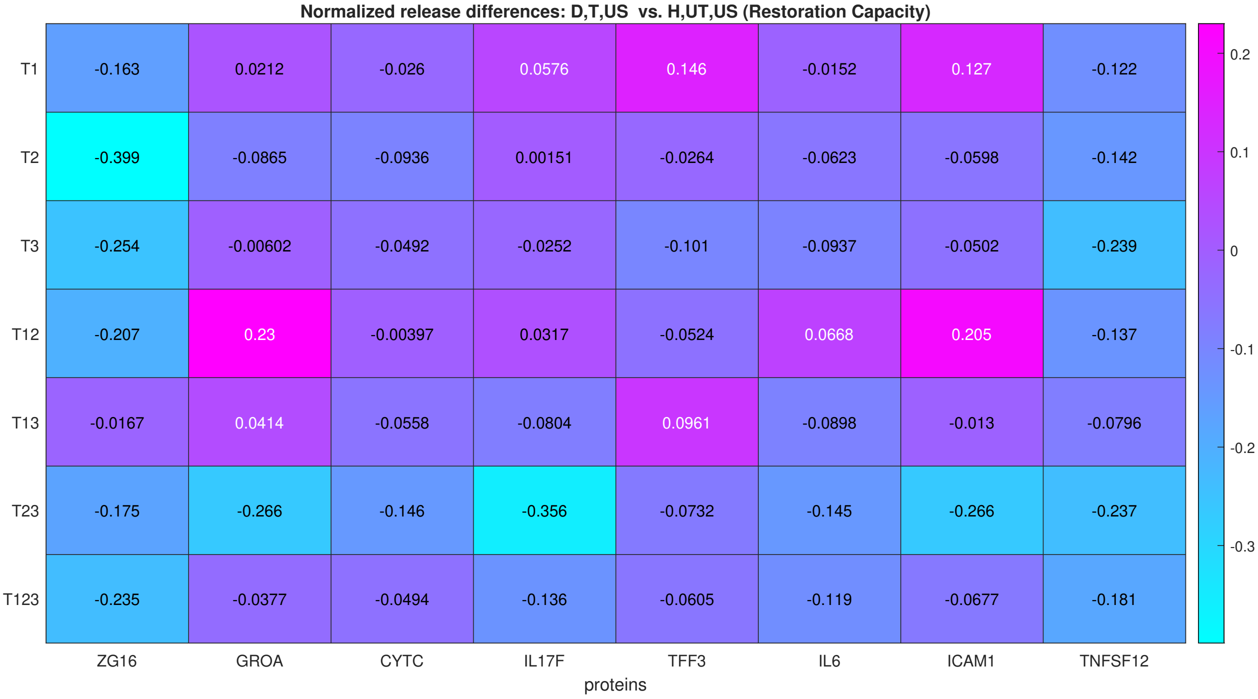

Figure S7: Normalized release differences between D,treated (T),unstimulated (US) and H,untreated (UT),unstimulated cells (US). A particular row corresponds to a particular treatment T_x and shows how far from normal are the T_x-induced protein releases. For example, T_{13} seems to have induced the most similar protein releases to healthy cells of all other treatments; $T_1, T_2, T_{12}, T_{23}, T_{123}$.

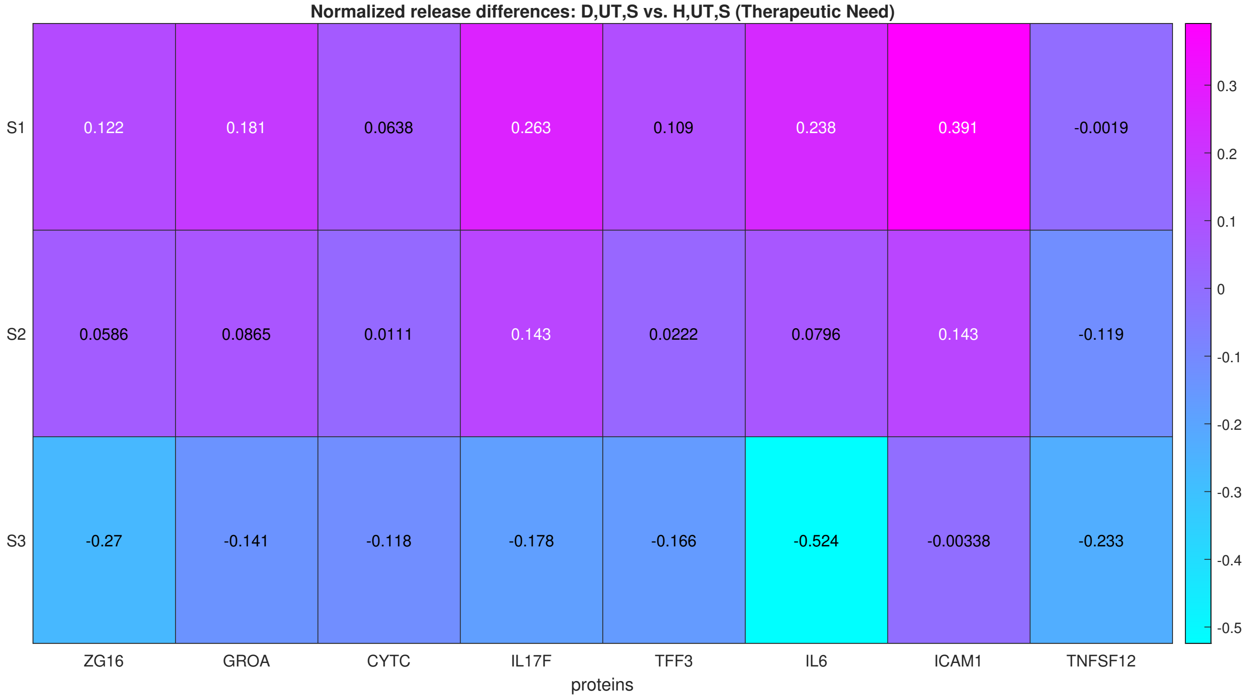

Figure S8: Normalized release differences between D,untreated (UT),stimulated (S) and H,untreated (UT),stimulated (S) cells. Each row corresponds to a particular stimulation (S_1, S_2, S_3). Compared to the corresponding normalized protein release differences for the unstimulated cells (Fig. S5), stimulating with S_1 and S_3 is associated with noticeable changes for several proteins.

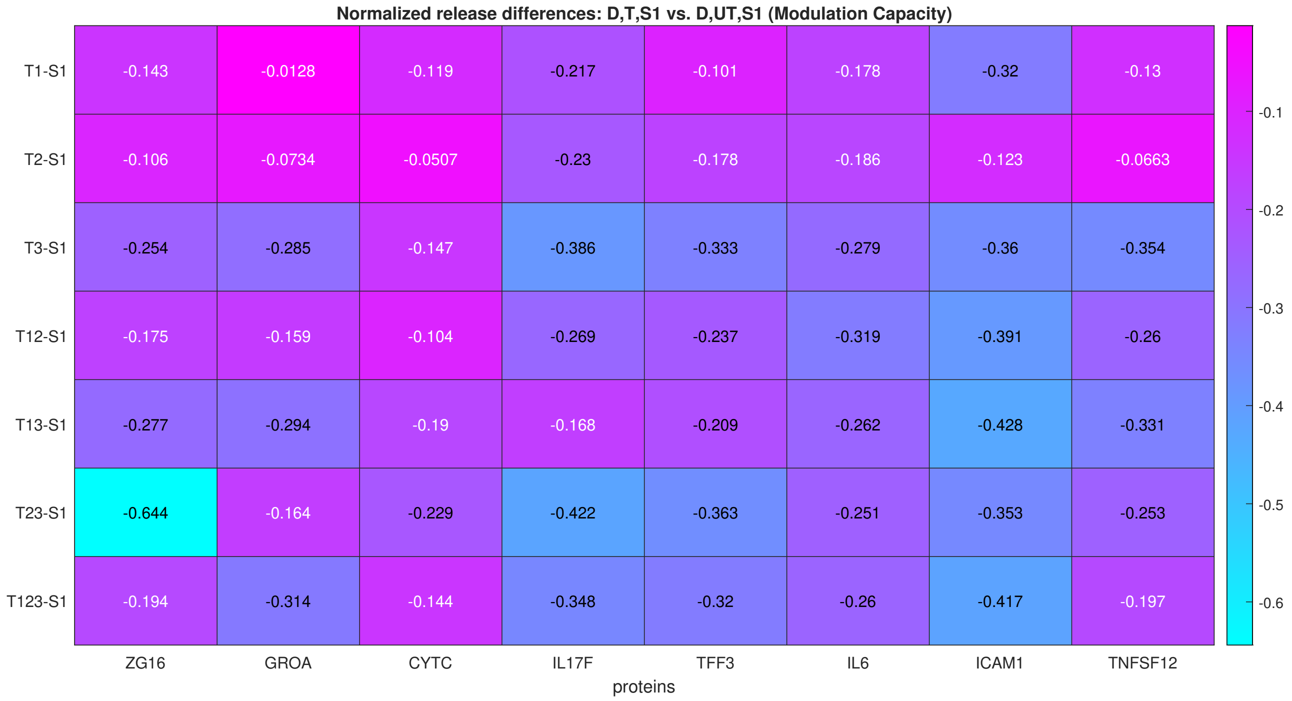

Figure S9: Normalized release differences between D,treated (T),stimulated with S1 and D,untreated (UT),stimulated with S1 cells. A particular row shows how much a particular treatment T_x has affected the protein releases of D cells after being stimulated with the S_1. Focusing on protein ZG16 and treatment T_{23}, the corresponding unstimulated release (Fig. S6) dropped only $\approx 9\%$, while the release after stimulation with S_1 decreased $\approx 64\%$.

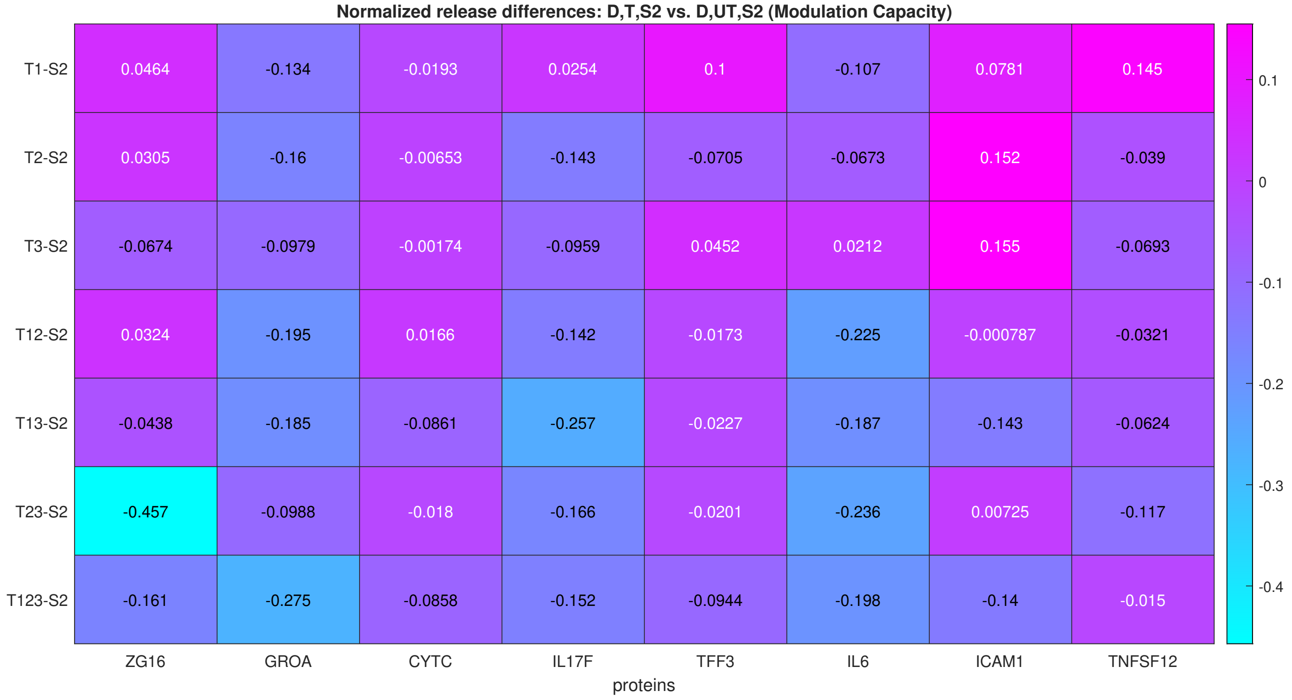

Figure S10: Normalized release differences between D,treated (T),stimulated with S2 and D,untreated (UT),stimulated with S2 cells. A particular row shows how much a particular treatment T_x has affected the protein releases of D cells after being stimulated with the S_2.

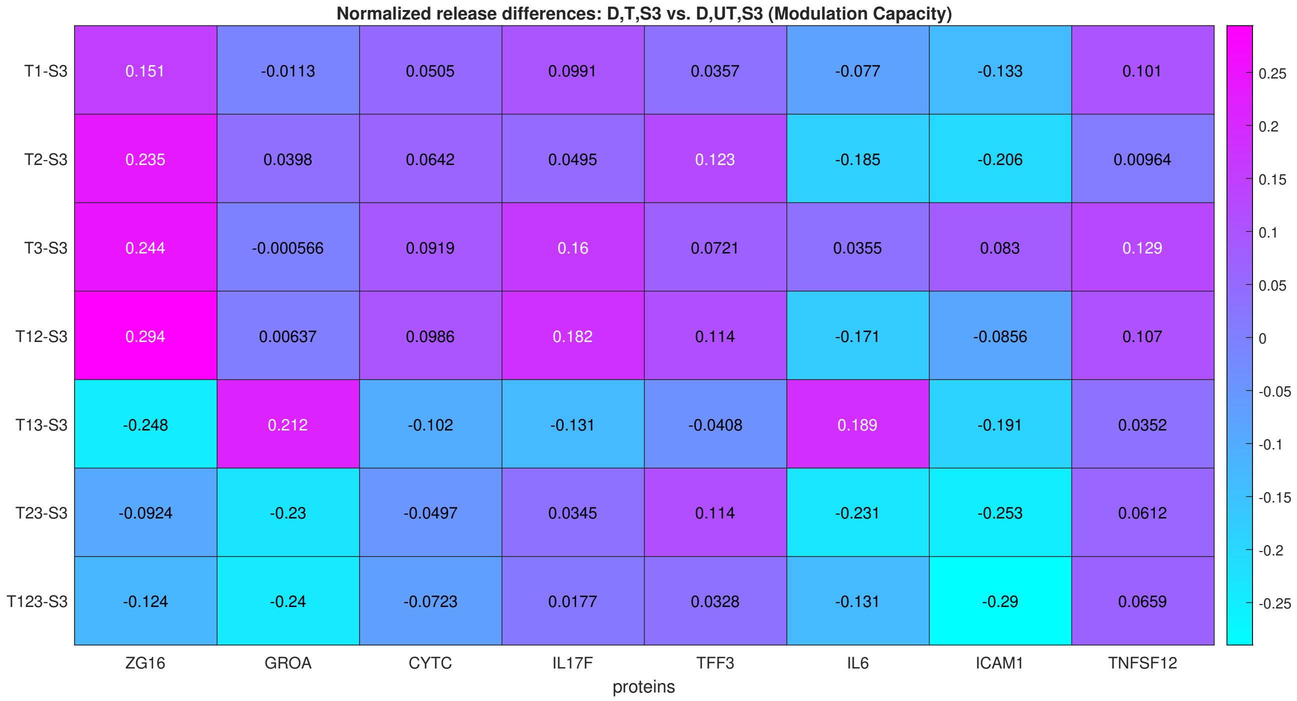

Figure S11: Normalized release differences between D,treated (T),stimulated with S3 and D,untreated (UT),stimulated with S3 cells. A particular row shows how much a particular treatment T_x has affected the protein releases of D cells after being stimulated with the S_3.

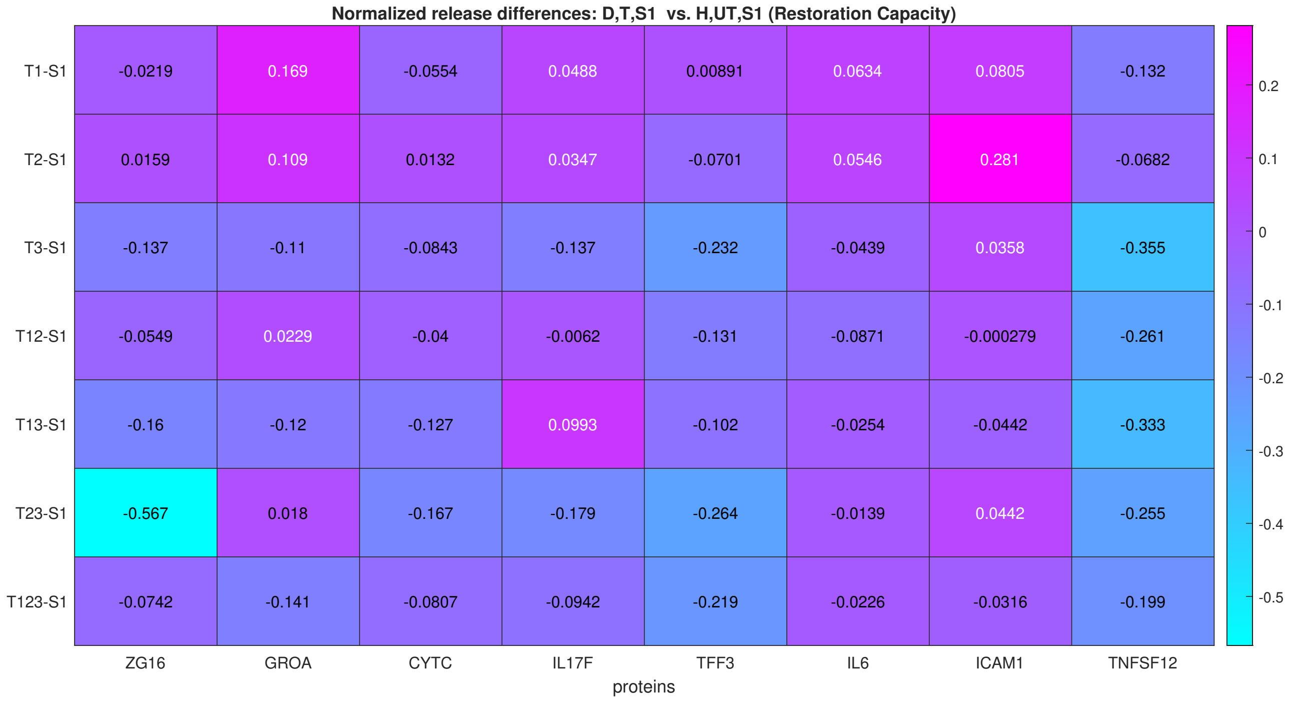

Figure S12: Normalized release differences between D,treated (T),stimulated with S1 and H,untreated (UT),stimulated with S1 cells. A particular row corresponds to a particular treatment T_x and shows how far from normal are the T_x-induced protein releases after stimulation with $S1$. Focusing on protein $ZG16$ and treatment T_{23}, there was $\approx 17\%$ drop when cells remained unstimulated (Fig. S7), while after stimulation with S_1 the release of ZG16 decreased $\approx 57\%$. However, there are examples towards the opposite direction, such as T_2, where the release of ZG16 dropped much more for the unstimulated cells ($\approx 40\%$) than the S_1-stimulated cells ($\approx 1.6\%$).

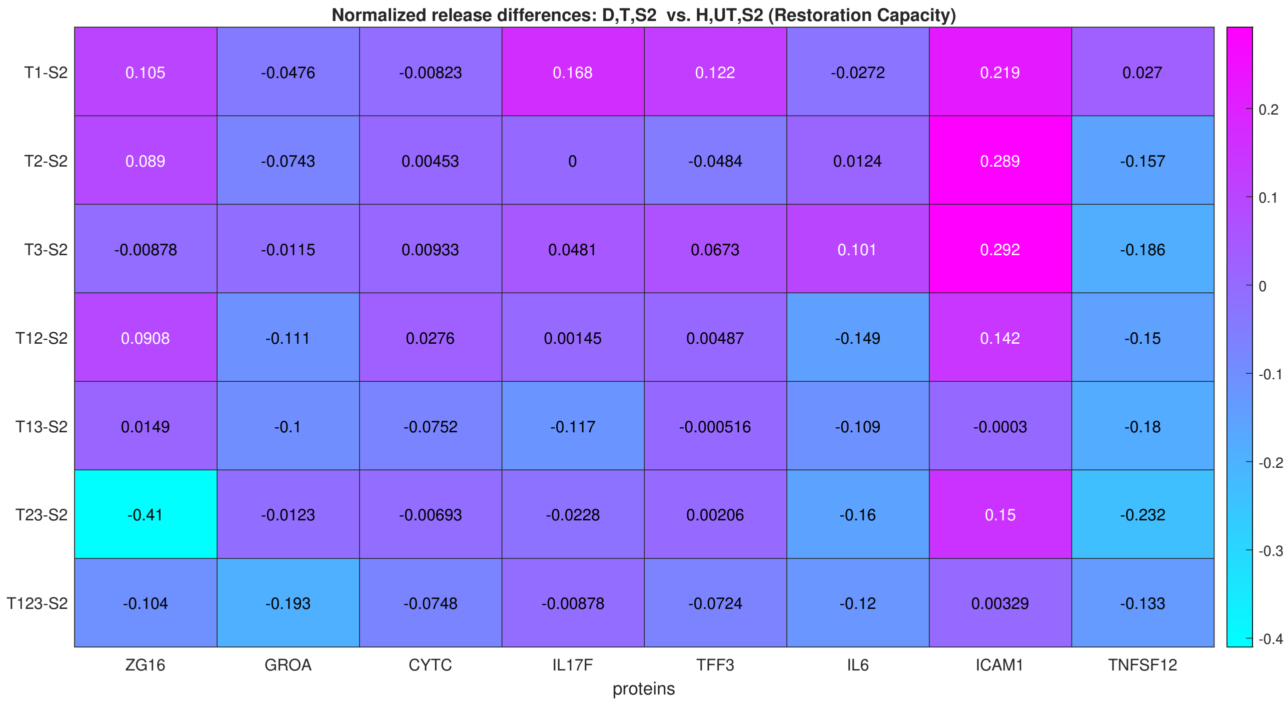

Figure S13: Normalized release differences between D,treated (T),stimulated with S2 and H,untreated (UT),stimulated with S2 cells. A particular row corresponds to a particular treatment T_x and shows how far from normal are the T_x-induced protein releases after stimulation with $S2$.

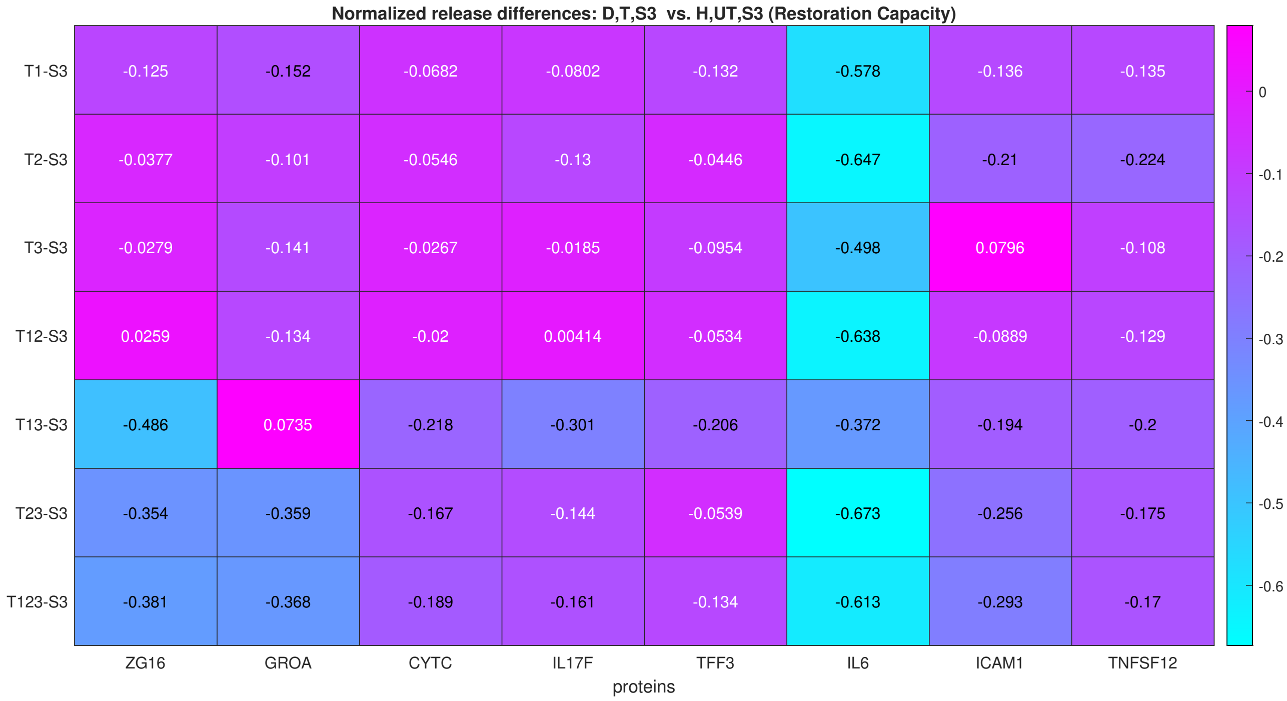

Figure S14: Normalized release differences between D,treated (T),stimulated with S2 and H,untreated (UT),stimulated with S2 cells. A particular row corresponds to a particular treatment T_x and shows how far from normal are the T_x-induced protein releases after stimulation with $S3$.

a

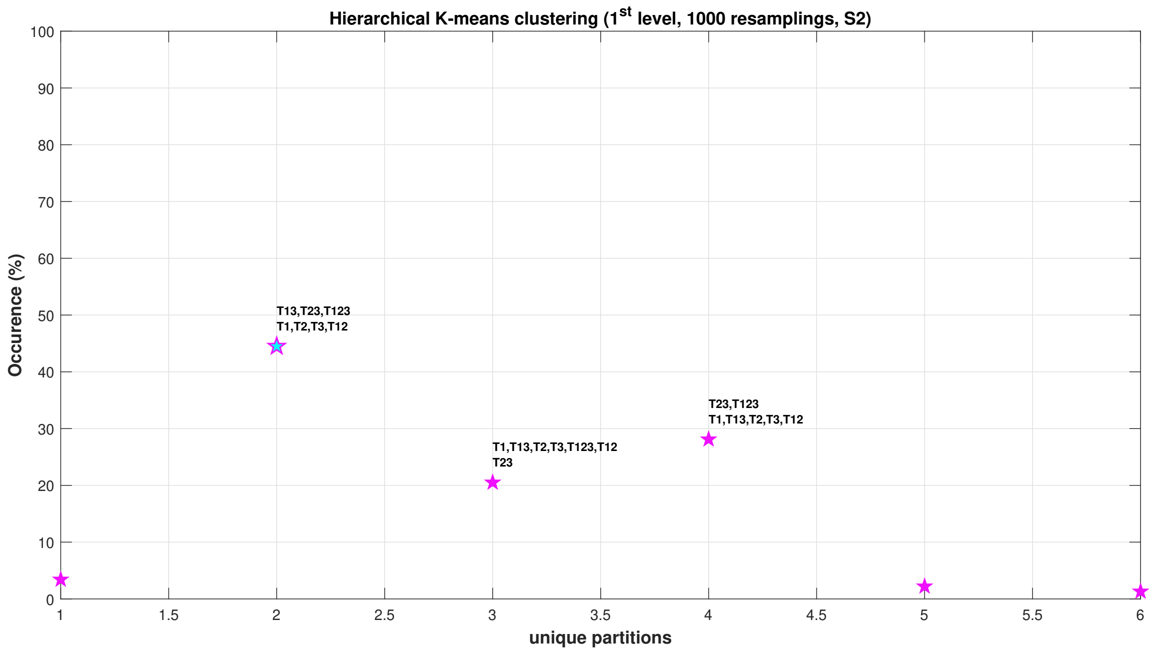

b

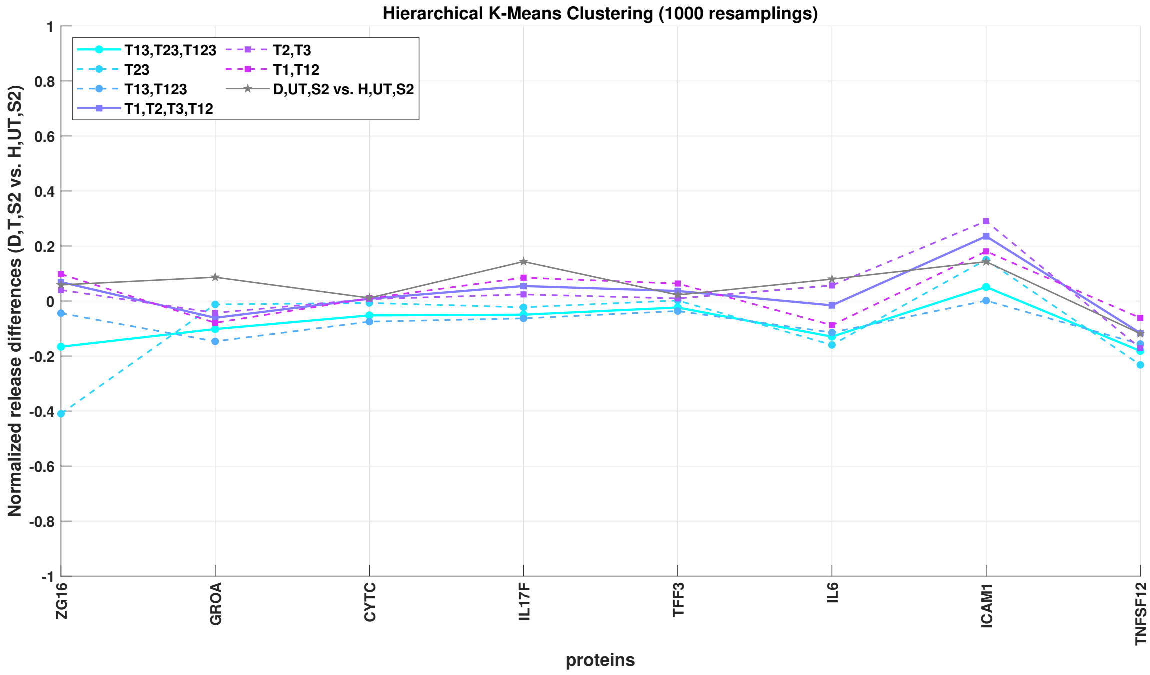

Figure S15: Top-down hierarchical K-Means clustering for stimulation S_2 and $N_v = 10^3$ validation datasets. **(a)** Frequency/occurrence (%) for all unique partitions/clusters at the first hierarchical level. Annotations are provided for the three most frequent partitions. **(b)** Visualization of the clustering results after validation; the most dominant partitions at the first and second hierarchical levels are used. Each line corresponds to the centroid of each (sub-)cluster identified representing its restoration capacity defined by the normalized protein release differences between D,treated,stimulated with S_2 (D, T, S_2) and H,untreated,stimulated with S_2 (H, UT, S_2) cells. Solid and dotted lines are used for the first and second hierarchical level respectively. The gray solid line corresponds to the total therapeutic need, meaning the normalized protein release differences between D,untreated,stimulated with S_2 (D, UT, S_2) and H,untreated,stimulated with S_2 (H, UT, S_2) cells, which an ideal treatment should eradicate. The legend shows all treatments per (sub-)cluster identified without employing exhaustive subset search since only 7 treatments were used in total.

a

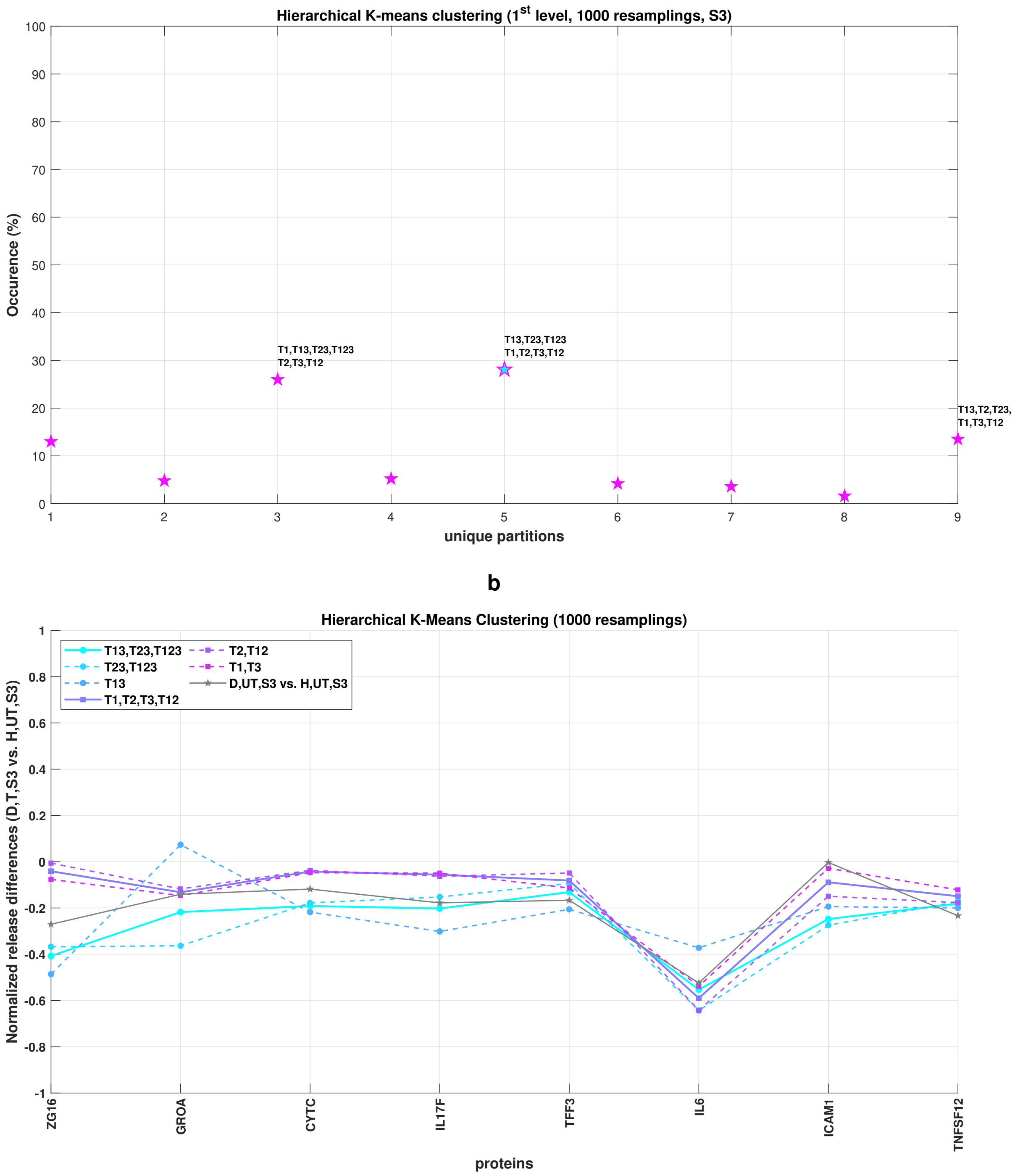

Figure S16: Top-down hierarchical K-Means clustering for stimulation S_3 and $N_v = 10^3$ validation datasets. **(a)** Frequency/occurrence (%) for all unique partitions/clusters at the first hierarchical level. Annotations are provided for the three most frequent partitions. **(b)** Visualization of the clustering results after validation; the most dominant partitions at the first and second hierarchical levels are used. Each line corresponds to the centroid of each (sub-)cluster identified representing its restoration capacity defined by the normalized protein release differences between D,treated,stimulated with S_3 (D, T, S_3) and H,untreated,stimulated with S_3 (H, UT, S_3) cells. Solid and dotted lines are used for the first and second hierarchical level respectively. The gray solid line corresponds to the total therapeutic need, meaning the normalized protein release differences between D,untreated,stimulated with S_3 (D, UT, S_3) and H,untreated,stimulated with S_3 (H, UT, S_3) cells, which an ideal treatment should eradicate. The legend shows all treatments per (sub-)cluster identified without employing exhaustive subset search since only 7 treatments were used in total.

a

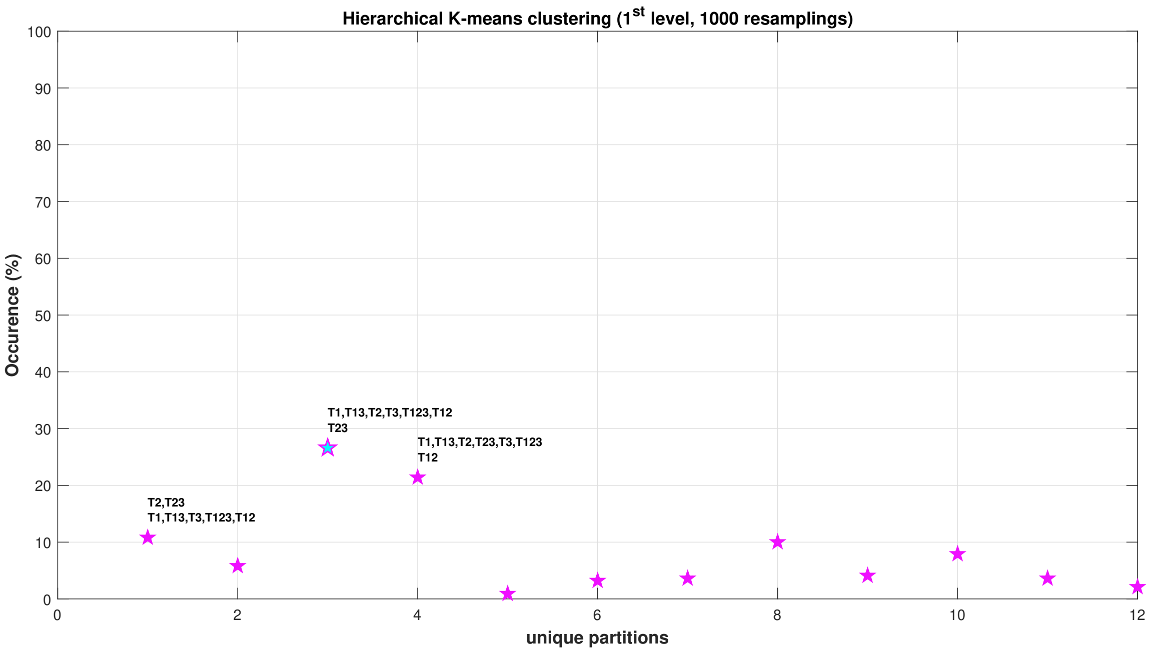

b

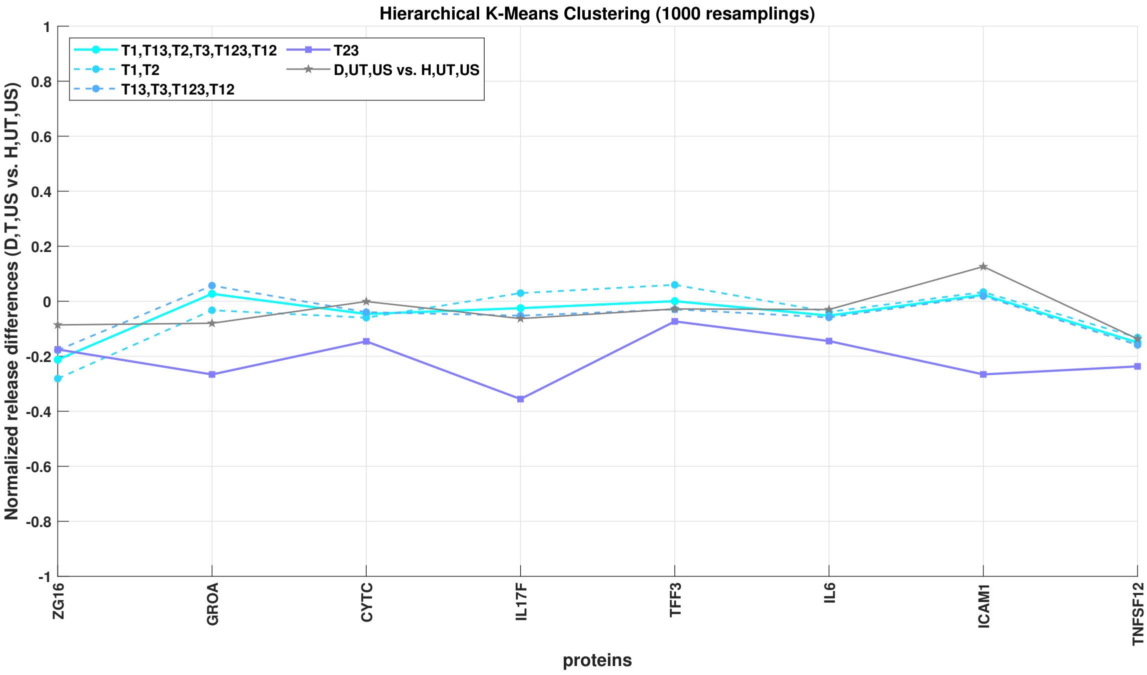

Figure S17: Top-down hierarchical K-Means clustering for unstimulated cells and $N_v = 10^3$ validation datasets. **(a)** Frequency/occurrence (%) for all unique partitions/clusters at the first hierarchical level. Annotations are provided for the three most frequent partitions. **(b)** Visualization of the clustering results after validation; the most dominant partitions at the first and second hierarchical levels are used. Each line corresponds to the centroid of each (sub-)cluster identified representing its restoration capacity defined by the normalized protein release differences between D,treated,unstimulated (D, T, US) and H,untreated,unstimulated (H, UT, US) cells. Solid and dotted lines are used for the first and second hierarchical level respectively. The gray solid line corresponds to the total therapeutic need, meaning the normalized protein release differences between D,untreated,unstimulated (D, UT, US) and H,untreated,unstimulated (H, UT, US) cells, which an ideal treatment should eradicate. The legend shows all treatments per (sub-)cluster identified without employing exhaustive subset search since only 7 treatments were used in total.

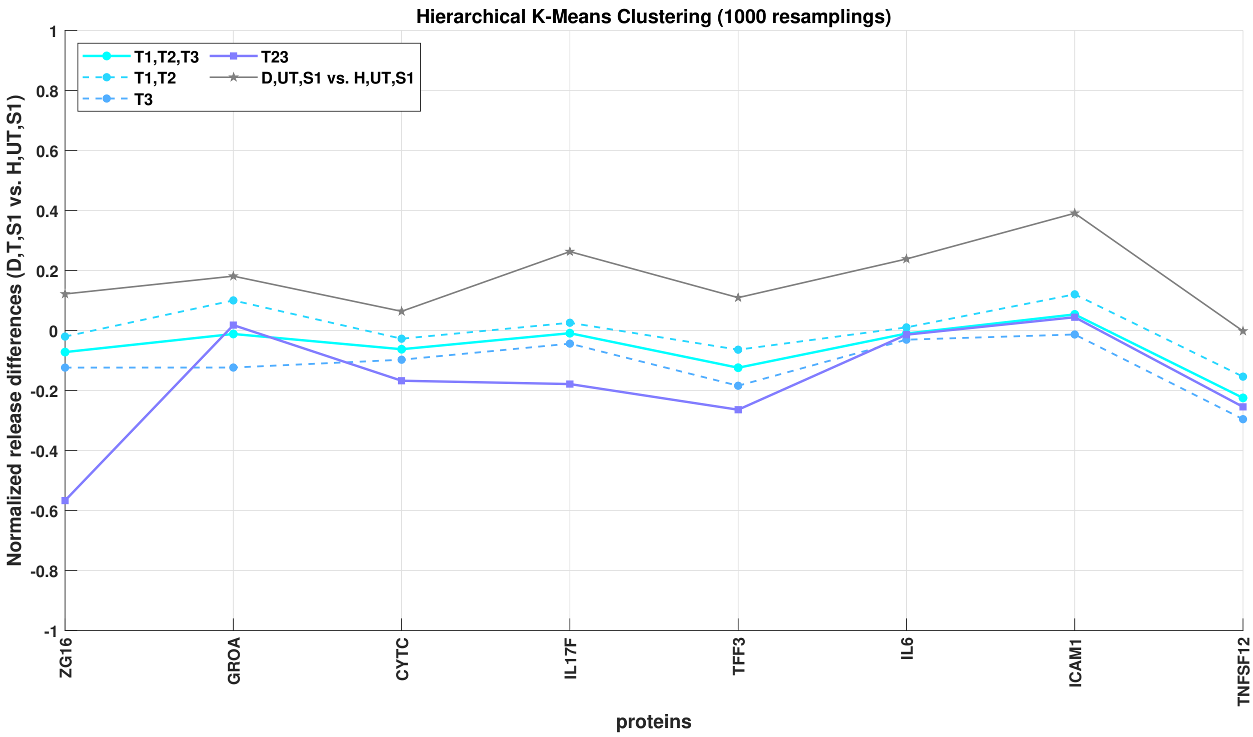

Figure S18: Top-down hierarchical K-Means clustering for stimulation S_1 and $N_v = 10^3$ validation datasets using exhaustive subset search. Visualization of the clustering results after validation; the most dominant partitions at the first and second hierarchical levels are used. Each line corresponds to the centroid of each (sub-)cluster identified representing its restoration capacity defined by the normalized protein release differences between D,treated,stimulated with S_1 (D, T, S_1) and H,untreated,stimulated with S_1 (H, UT, S_1) cells. Solid and dotted lines are used for the first and second hierarchical level respectively. The gray solid line corresponds to the total therapeutic need, meaning the normalized protein release differences between D,untreated,stimulated with S_1 (D, UT, S_1) and H,untreated,stimulated with S_1 (H, UT, S_1) cells, which an ideal treatment should eradicate. The difference with Fig. 5b in the main article text is that the legend has been created using the exhaustive subset search option and thus, it only contains the unique treatments that induce the prototypical protein response profiles for each (sub-)cluster identified.

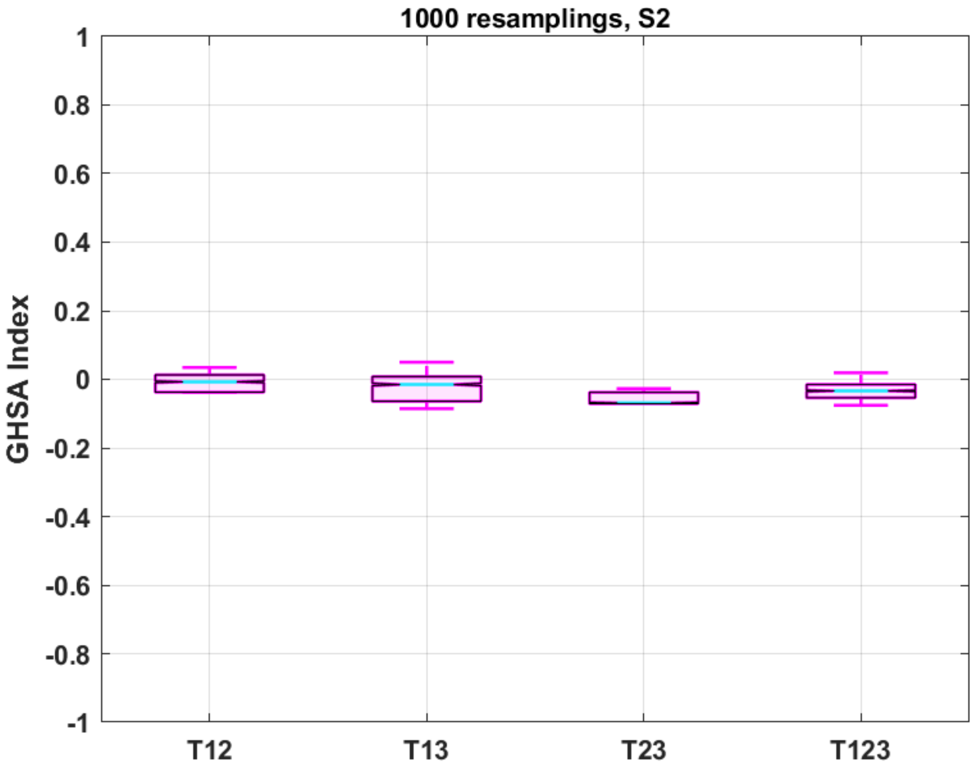

Figure S19: GHSA analysis for stimulation S_2 and $N_v = 10^3$ validation datasets. All four combination treatments $T_{12}, T_{13}, T_{23}, T_{123}$ are shown on the x-axis. Each combination treatment is represented by a box plot showing the minimum value, $25^{th}, 50^{th}, 75^{th}$ percentiles and maximum value of all N_v GHSA indices obtained during a resampling-based leave-one-out validation approach.

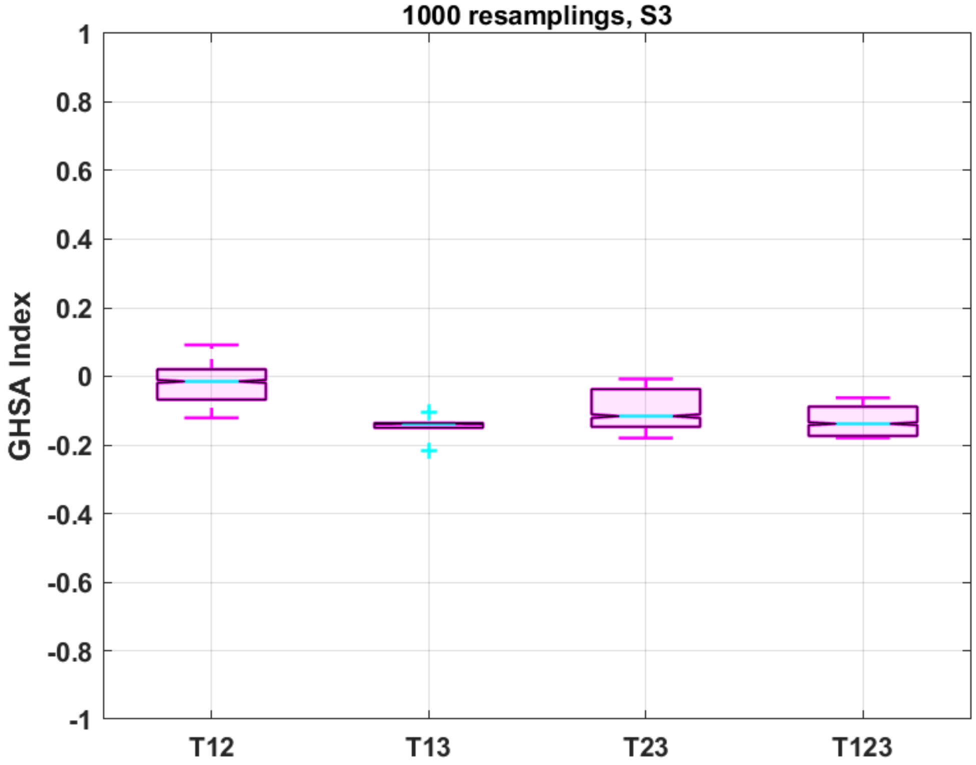

Figure S20: GHSA analysis for stimulation S_3 and $N_v = 10^3$ validation datasets. All four combination treatments $T_{12}, T_{13}, T_{23}, T_{123}$ are shown on the x-axis. Each combination treatment is represented by a box plot showing the minimum value, $25^{th}, 50^{th}, 75^{th}$ percentiles and maximum value of all N_v GHSA indices obtained during a resampling-based leave-one-out validation approach.

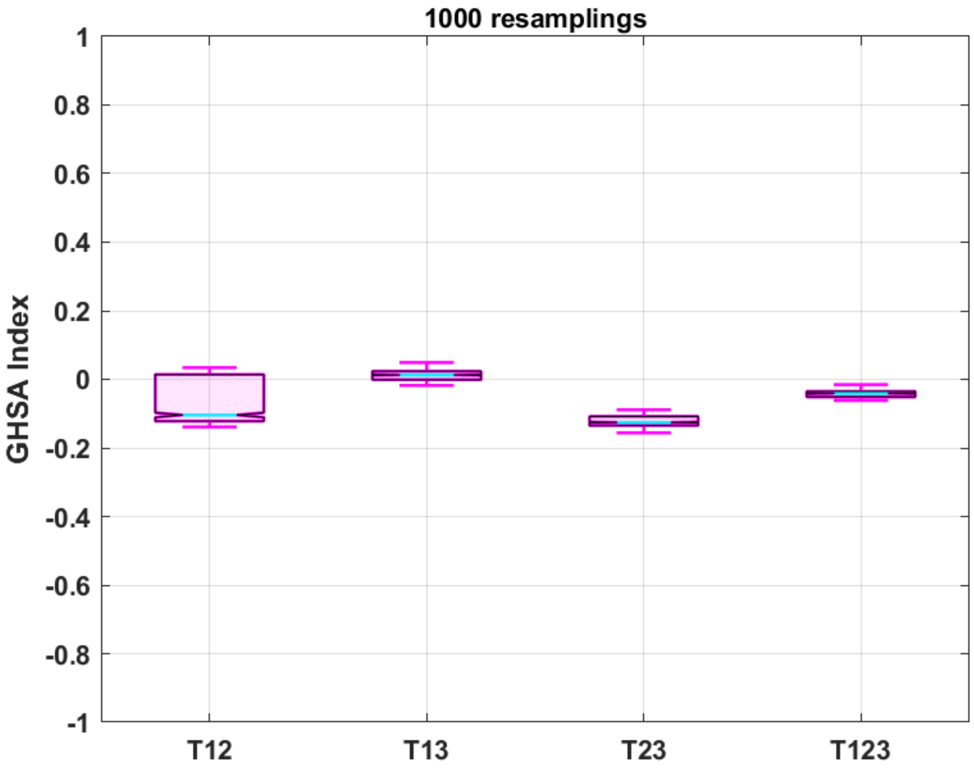

Figure S21: GHSA analysis for unstimulated cells and $N_v = 10^3$ validation datasets. All four combination treatments $T_{12}, T_{13}, T_{23}, T_{123}$ are shown on the x-axis. Each combination treatment is represented by a box plot showing the minimum value, $25^{th}, 50^{th}, 75^{th}$ percentiles and maximum value of all N_v GHSA indices obtained during a resampling-based leave-one-out validation approach.

3 Example raw data file

In this section, instructions on how to provide a valid data file (i.e., file with raw protein release measurements) are provided. The raw data file must be in CSV file format using the barcode of the microtiter plate as the corresponding filename ($< barcode.csv >$). Its content should be structured as described below. The first column and row should contain annotations of the collected dataset, namely the cell state of experimental wells and measured protein releases. The rest of rows and columns correspond to the numeric part of the dataset, which is stored in the form of a $N \times d$ matrix. N and d denote the number of total wells and measured proteins per experimental well, respectively. In the example raw data file, $N = 9$ and $d = 3$. More precisely, each row corresponds to a particular experimental well and contains the raw release measurement values for proteins PEDF, CXCL11 and IL13. The cell state of a particular experimental well is described in column "Sample" of the corresponding row.

COMBsecretomics requires the following naming convention for the cell states included in column "Sample". Blank wells should be declared as "BLANK" or "blank". For wells containing cells, a three-field naming convention is required in the following form:

$$< Cells > - < Treatment > - < Stimulation >.$$

The field $< Cells >$ should contain an identifier related to the type of cells in a particular well. For example, here we have used D for disease releated but the user is free to choose this field. Healthy cells must be denoted H. The field $< Treatment >$ must be either set to "UT" for untreated cells or "TX" for cells being treated with TX, where X must consist of the numeric identifier for a particular treatment. For instance, $T1$ corresponds to the treatment consisting

of compound 1, $T12$ corresponds to the combination treatment consisting of compounds 1 and 2, while $T123$ corresponds to the combination treatment consisting of compounds 1, 2 and 3. Therefore, the user has to annotate the treatments using numbers instead of names. Finally, the field *Stimulation* must be either set to “US” for unstimulated cells or “SY” for stimulated cells, where Y declares the numeric identifier of the corresponding stimulation. For example, $S1$ corresponds to stimulation 1, $S2$ corresponds to stimulation 2 etc. Similarly, the user has to annotate the stimulations using numbers instead of descriptive names (see also https://github.com/EffieChantzi/COMBSecretomics.git).

Example raw data file

Sample	PEDF	CXCL11	IL13
D-UT-US	708	543	353
D-T1-US	753	375	428
D-T12-US	685	521.5	513
D-T123-US	420.5	408	293.5
D-T1-S1	513	357	378.5
D-T12-S1	495	408	630.5
D-T123-S1	9251	496	588
H-UT-US	15274	455	487.5
H-UT-S1	859	328	539.5
BLANK	636	296.5	329

4 User-defined inputs

In this section, recommendations on how to select the user-defined inputs to COMBSecretomics are provided (see also https://github.com/EffieChantzi/COMBSecretomics.git):

(1) Raw data file is selected interactively by the user. Details on how to provide a valid raw data file with the collected protein release data and the corresponding annotations are given in the previous section “Example raw data file”.

(2) Cut-off threshold (%) for the blank filtering, as part of the quality control (see section “Blank filtering”, above). By setting a low value, one ensures that proteins with low levels of noise are kept for further analysis.

(3) Cut-off threshold (%) for the coefficient of variation for the measured protein releases, as part of the quality control (see section “Coefficient of variation”, above). By setting a low value, one ensures that proteins with low levels of technical variability are kept for further analysis.

(4) Number of resampling based validation datasets to be created (see section “Resampling statistics”, main article text). The higher this number, the more datasets are going to be created for validation, which is advisable especially if several intra-plate replicate measurements are included in the experimental design. Here, the trade-off between quality and quantity should be taken into account. It might be that few obtained results appear to be stable but these few are likely to be reproducible. Despite being experimentally expensive, multiple replicate measurements are highly recommended for this type of validation approaches.

(5) Option for employing exhaustive subset search when visualizing the combination analysis results from the hierarchical clustering (see section “Top-down hierarchical clustering”, main article text). We highly recommend this option, especially for large exhaustive experimental set ups, as it provides a very helpful way of disentangling higher- from lower- and single-order treatment effects and summarizing the prototypical chemically induced protein release patterns.

(6) Analysis of protein release measurements for unstimulated and stimulated cells. The user is advised to run the analyses for both types of cells sequentially.
